# Reproducibility of brachial artery flow-mediated and glyceryl trintrate-mediated dilatation by 3Tesla CMR

**DOI:** 10.1186/1532-429X-14-S1-P292

**Published:** 2012-02-01

**Authors:** James Oliver, Peter Swoboda, Sven Plein, Kate English, George Ballard, John P Greenwood

**Affiliations:** 1 Leeds Institute of Genetics, Health and Therapeutics, University of Leeds, Leeds, UK; 2 Cardiology, Leeds Teaching Hospitals, Leeds, UK

## Summary

Intra-observer and inter-study reproducibility of brachial artery flow-mediated dilatation (FMD) and glyceryl trintrate (GTN)-mediated dilatation by CMR at 3Tesla were assessed. Coefficients of variation (CoV) were calculated using the root mean square method. The intra-observer CoV, assessed in 15 subjects, was 15% for both FMD and GTN response. The inter-study CoV, assessed in 17 subjects, was 24% for FMD and 34% for GTN response.

## Background

Flow-mediated dilatation (FMD) of the brachial artery is most commonly measured using ultrasound. However, CMR can also be used to measure FMD. The aim of this study was to assess the intra-observer and inter-study reproducibility of FMD measured by CMR at 3Tesla.

## Methods

CMR was performed on a Philips Achieva 3.0T TX scanner. Brachial arterial reactivity was assessed by high spatial resolution cine imaging (0.2x0.2mm in plane) perpendicular to the artery 5cm above the elbow. After a baseline acquisition a cuff around the forearm was inflated to 50mmHg above systolic blood pressure for 5 minutes. Following cuff deflation further images were acquired after 15, 30, 45, 60 and 75 seconds. 10 minutes after cuff deflation a 2nd baseline image of the artery was acquired and 25mcg of glyceryl trinitrate (GTN) administered sublingually. Additional images were then acquired every 20 seconds from 2 to 5 minutes after drug administration.

End-diastolic brachial artery area at each time point was determined by manual tracing in Qmass 7. 2 (Medis, The Netherlands). The FMD response was calculated as the maximum percentage increase in artery area in the 75 seconds following cuff release. The response to GTN was calculated as the maximum percentage increase in artery area from the pre-GTN baseline.

The root mean square method was used to calculate coefficients of variation (CoVs) for FMD and GTN response. The data from the 17 patients who underwent 2 scans was used to assess inter-study reproducibility. Of the total of 44 scans 15 were selected at random to be reanalysed for intra-observer reproducibility.

## Results

The results are illustrated in the Table.

**Table 1:** 

	Intra-observer			Inter-study		
	Analysis 1	Analysis 2	CoV(%)	Study 1	Study 2	CoV(%)

FMD (%)	16.0 (7.5)	15.6 (7.3)	15 (21)	15.8 (5.5)	16.2 (6.4)	24 (34)
GTN response (%)	26.9 (9.7)	27.2 (11)	15 (19)	35.1 (12.5)	29 (9.8)	34 (41)

Means (SDs) and CoVs (upper 95% confidence limit) for FMD and GTN response.

## Conclusions

There is reasonably good intra-observer reproducibility in brachial artery FMD and GTN response as measured by CMR. Inter-study variability in these measures is quite substantial. These data will inform sample size calculations for future clinical studies.

## Funding

Dr Oliver's post is funded by the UK National Institute for Health Research. This work was supported by a Clinical Lecturer Starter Grant from the UK Academy of Medical Sciences.

